# Insights into the Development and Evolution of Exaggerated Traits Using *De Novo* Transcriptomes of Two Species of Horned Scarab Beetles

**DOI:** 10.1371/journal.pone.0088364

**Published:** 2014-02-20

**Authors:** Ian A. Warren, J. Cristobal Vera, Annika Johns, Robert Zinna, James H. Marden, Douglas J. Emlen, Ian Dworkin, Laura C. Lavine

**Affiliations:** 1 Department of Entomology, Washington State University, Pullman, Washington, United States of America; 2 School of Biological Sciences, University of Bristol, Bristol, United Kingdom; 3 Department of Biology, Pennsylvania State University, State College, Pennsylvania, United States of America; 4 Division of Biological Sciences, The University of Montana, Missoula, Montana, United States of America; 5 Program in Ecology, Evolutionary Biology and Behavior, Michigan State University, East Lansing, Michigan, United States of America; 6 Department of Zoology, Michigan State University, East Lansing, Michigan, United States of America; University of Lausanne, Switzerland

## Abstract

Scarab beetles exhibit an astonishing variety of rigid exo-skeletal outgrowths, known as “horns”. These traits are often sexually dimorphic and vary dramatically across species in size, shape, location, and allometry with body size. In many species, the horn exhibits disproportionate growth resulting in an exaggerated allometric relationship with body size, as compared to other traits, such as wings, that grow proportionately with body size. Depending on the species, the smallest males either do not produce a horn at all, or they produce a disproportionately small horn for their body size. While the diversity of horn shapes and their behavioural ecology have been reasonably well studied, we know far less about the proximate mechanisms that regulate horn growth. Thus, using 454 pyrosequencing, we generated transcriptome profiles, during horn growth and development, in two different scarab beetle species: the Asian rhinoceros beetle, *Trypoxylus dichotomus*, and the dung beetle, *Onthophagus nigriventris*. We obtained over half a million reads for each species that were assembled into over 6,000 and 16,000 contigs respectively. We combined these data with previously published studies to look for signatures of molecular evolution. We found a small subset of genes with horn-biased expression showing evidence for recent positive selection, as is expected with sexual selection on horn size. We also found evidence of relaxed selection present in genes that demonstrated biased expression between horned and horn-less morphs, consistent with the theory of developmental decoupling of phenotypically plastic traits.

## Introduction

Evolution has generated a spectacular array of secondary sexually selected traits [1], with the most well known examples including the peacock's train [2], antlers on cervids [3], and brightly coloured dewlaps of the anole lizard [4]. These exaggerated traits tend to be most prominent in males and can affect female preference, or be used as weapons to determine the outcome of male-male conflict. Expression of exaggerated, sexually selected traits tends to be closely correlated with male condition [5]. Smaller males either grow vastly reduced traits, or they fail to grow the trait entirely [6], in which case they often adopt alternative mating strategies, such as “sneaker matings”, where non-dominant males attempt matings without the knowledge of the dominant males [7].

Evolutionary processes favouring the emergence and subsequent maintenance of exaggerated sexually selected traits have been discussed for over a century and studied in a diverse range of species [1], [8], [9]. Far less is known about the genetic mechanisms that facilitate extreme growth of these traits, and how regulation of exaggerated trait development has evolved. Several key issues need to be addressed, including: understanding how exaggerated growth of traits arise, and how final trait size is modulated in response to the physiological condition of males [10]; as well as understanding how exaggerated traits are developmentally related to other traits, which is especially important when exaggerated structures are also novel structures (e. g. what common pathways have been co-opted into the development of the novel trait?) [11], [12].

**Figure 1:**
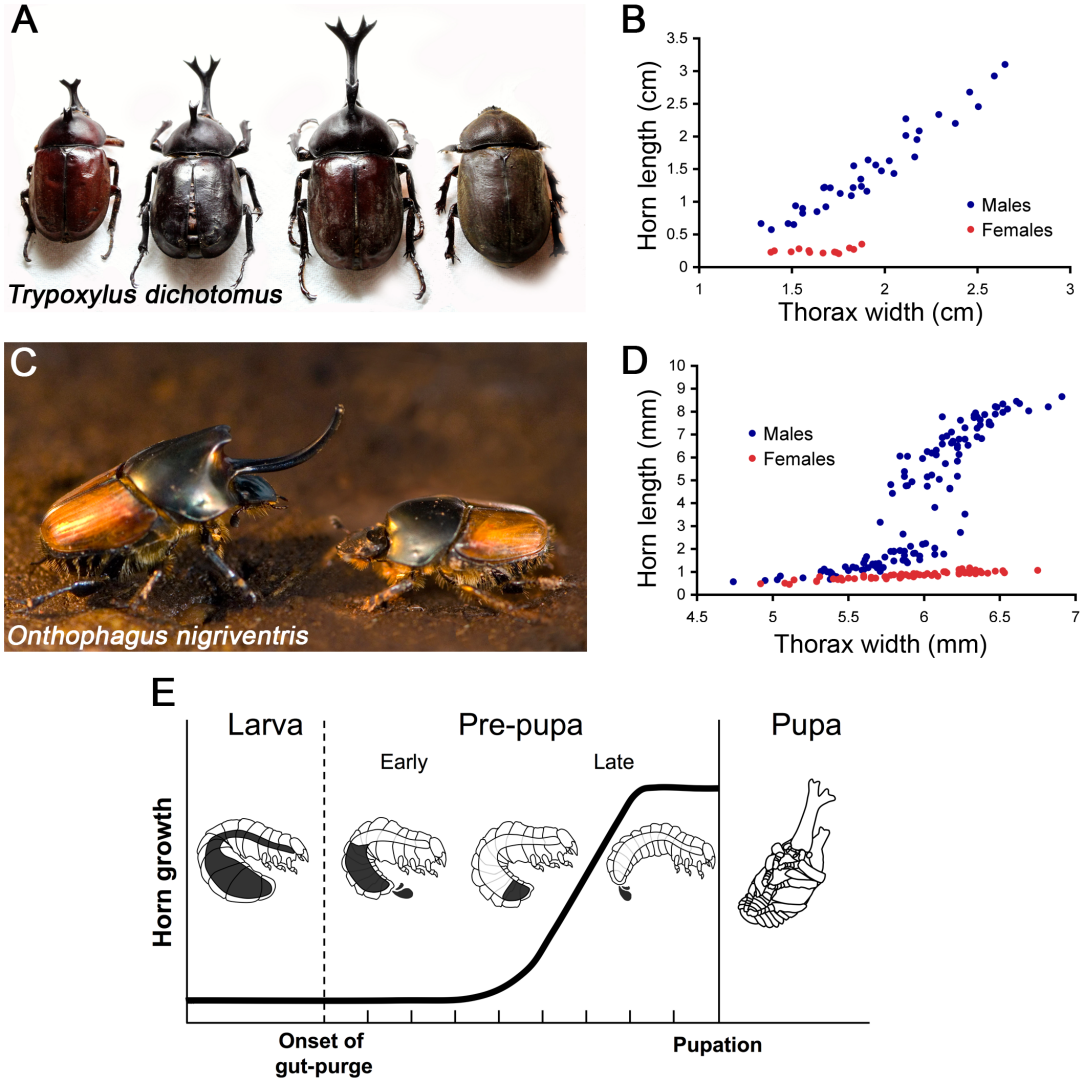
Horned beetles and horn growth. (A) Three *T. dichotomus* males (left) with small increases in body size, associated with more dramatic increases in horn size. Males can be up to 7 cm in length (20 g), with a forked horn protruding anteriorly from the head that can be upto 4.5 cm in length. *T. dichotomus* females (far right) bear no exaggerated horn (photo: A. Johns). Scale bar  = 1 cm (B) Continuous relationship of head horn size and body size in T. dichotomus. Thorax width is used as a proxy for body-size (for justification see [20], [74]). (C) Large *O. nigriventris* males (left) are 10–12 mm in length (25 mg) and exhibit a large thoracic horn that can reach upto 16 mm. Females and small males (right) do not grow exaggerated thoracic horns (photo: D. Emlen). Scale bar  = 5 mm (D) Relationship of body size and thoracic horn length in *O. nigriventris*. (E) A schematic of development during the period prior to pupation, a critical period in horn growth. Late in the third larval instar the larva stops feeding and undergoes gut purge, and becomes a “pre-pupa”. Once the gut purge is complete, the larva undergoes pupation. Head horn imaginal discs form underneath the head capsule, late in the third larval instar. During the pre-pupal period, these imaginal discs undergo intense growth, with horns becoming clearly visible in the pupa (A. Johns unpublished data).

The scarab beetles (Coleoptera: Scarabaeidae) are a model taxonomic group for answering these questions [13], [14]. Many of the more than 30,000 species within this superfamily of beetles possess rigid exo-skeletal outgrowths, known as “horns”, which are often highly exaggerated (Fig. 1). Beetle horns are used as weapons to settle male-male disputes for access to females [15], [16] and appear to have arisen independently on multiple occasions within this clade [6]. There is immense diversity in horn morphology between species, including differences in horn shape, location, number, and allometry with body size [6], [17], [18]. There is also considerable variation in horn morphology within species [6], [18]. Sexual dimorphism is common, typically with males bearing the trait and females either lacking the trait or producing a much smaller structure. Intra-sexual nutrition-dependent size variation is frequent: males that receive high levels of larval food grow large body sizes coupled with disproportionately large horns, whereas males that receive poor larval nutrition exhibit reduced horns or, in some species, do not grow horns at all [19]–[21]. Finally, inter-population differences in horn allometry with body size occur between wild populations [22], [23]. These characteristics of beetle horns make them particularly amenable as models for tackling important evolutionary questions regarding the evolution of novel traits, the development of extreme weapon size, shape, and location, and the mechanisms responsible for condition sensitive trait expression.

The candidate gene approach has proven successful for identifying genes involved in developmental patterning of the adult horn [24], as well as horn growth and proliferation [25], [26]. However, the candidate gene approach has major limitations, among them a strong bias towards examining the known functional role of genes from model systems, and the assumption that: A) these genes are also the most “important” ones in other species; and B) that the functional roles of particular genes are highly conserved across species.

An unbiased genomic approach is essential for understanding the complexity and richness of trait evolution in traditionally non-model systems. A set of expressed sequenced tags (ESTs) and subsequent micro-array studies in the scarab beetle, *Onthophagus taurus*, identified a number of genes likely to be involved in horn development [27], [28], including genes in pathways such as the insulin and juvenile hormone signalling pathways, as well as a number of patterning genes [27], [28]. Roche 454 pyrosequencing has been used to further characterise the transcriptome of *O. taurus* [29], but in order to fully understand what genes are important in horn growth, and how they have evolved in scarab beetles, it is necessary to sample multiple species across a broader range of the lineage.

To gain additional insight into the genetic underpinnings of scarab beetle horn development, we describe the 454 transcriptomes of two horned beetle species, *Trypoxylus* (formerly *Allomyrina*) *dichotomus* and *Onthophagus nigriventris* (Fig. 1). *T. dichotomus* (Coleoptera: Scarabaeidae: Dynastinae) (Fig. 1A) is commonly known as the Asian rhinoceros beetle and is native to East Asia. *T. dichotomus* displays sexually dimorphic horn expression, with males exhibiting a large forked head horn, along with an additional small horn on the pronotum [22] whereas females do not possess either horn (Fig. 1A&B). The natural history of this species has been especially well studied, including the behaviour of males and the functional role of horns in determining outcomes of contests over access to females [16], [17], [30]. In addition, the highly nutrition-sensitive nature of its horn growth has begun to be elucidated at the physiological level [16], [17], [30], [31].

Our second focal species, the dung beetle *O. nigriventris* (Coleoptera: Scarabaeidae: Scarabaeinae) (Fig. 1C), is part of the well-studied and speciose genus of scarab beetle, *Onthophagus* (>2000 species [6]). It is native to Africa, but also commonly found in Hawaii and Australia [32]. In *O. nigriventris* the candidate gene method has been applied to study horn growth [24], [26], [33], and several further candidate genes have been identified for this species using micro-arrays developed for the related beetle *O. taurus* [27], [28].

*Onthophagus nigriventris* is an outstanding species for comparison with *T. dichotomus* due to the nature of its horn growth. Large *O. nigriventris* males are capable of growing a large thoracic horn in a nutrition-dependent fashion whereas small males never produce a horn at all (Fig. 1C&D). The conspicuous male dimorphism of this species contrasts with *T. dichotomus*, which has an exaggerated head horn that shows a continuous and hyper-allometric relationship with male body size (Fig. 1A&B). We chose these two species because of their differences in horn location (head vs. thorax), horn shape (a forked horned versus a large spear), and their differing responses of horn growth to nutrition (continuous variation, vs. discrete variation e.g. presence/absence or “polyphenism”; Fig. 1). Finally, these species represent separate, independent evolutionary origins of exaggerated male weapons, arising from divergent subfamilies within the Scarabaeidae (Dynastinae and Scarabaeinae) that last shared a common ancestor over 100 million years ago [34], [35].

Using the transcriptome sequences generated here, we investigate the molecular evolution of genes involved in horn development from two contexts: sexual selection and developmental decoupling [28], [36], [37]. Intense sexual selection arising from male-male competition may drive more rapid evolution of genes influencing horn expression, resulting in signatures of recent positive selection. At the same time, not all individuals in a population express a horn (females and small males are horn-less in *O. nigriventris*, and females are horn-less in *T. dichotomus*) and therefore genes that are involved in horn development, over time, may be exposed to relatively weakened selection. This phenomenon is part of the theory of “developmental decoupling” [28], [36], [37]. In theory, both effects (intense selection for specific function and relaxed selection for general function) could occur simultaneously and may even interact, although positive selection via sexual selection may influence only a restricted subset of horn development genes. Genes with horn-biased gene expression and also morph-biased genes have previously been identified in *O. taurus*, but sufficient sequence data in closely related species were not available for robust estimates of selection. We used these *O. taurus* data to make comparisons with the two transcriptomes presented here. We detect evidence for both positive selection amongst genes with horn-biased expression, as well as evidence for relaxed purifying selection in genes showing morph-biased expression, consistent with theory and with previous observations [28], [38].

## Results

### Sequencing and assembly

We generated cDNA libraries from developing tissues in *Trypoxylus dichotomus* and *Onthophagus nigriventris*, and obtained over 750,000 raw reads for both species using Roche 454 pyrosequencing (Table 1). The sequencing approaches used for each species differed. Due to the large size of *T. dichotomus* it was possible to obtain total RNA from individual developing tissues, and thus libraries were generated for: head horn, thoracic horn, brain, genitals, fat body and forelegs from pre-pupal males (Fig. 1E); head, thorax and genitals from late 3^rd^ instar males (Fig. 1E); and head and thorax from late 3^rd^ instar females (Fig. 1E). This material and additional previously pooled RNA (from male developing pre-pupal tissues referred to as “library 12”, see methods) was used to make a total of 12 cDNA libraries. Due to its smaller size, such an approach was not possible for *O. nigriventris*. For this species mRNA was isolated from whole individual late 3^rd^ instar larvae and pre-pupae, which are pivotal growth periods for horn development (Fig. 1E). Prior to library construction and sequencing, both RNA and cDNA were checked for quality using high accuracy electropherograms (see methods). Both before and after quality filtering and trimming, the *O. nigriventris* sequencing run produced almost twice the number of reads as *T. dichotomus* (Table 1) with a mean read size almost 150 bp longer, probably due to differing sequencing strategies (see methods and discussion).

**Table 1:** Transcriptome assembly.

	*Trypoxylus dichotomus*	*Onthophagus nigriventris*
**Raw Reads**		
Number of reads	771,419	1,019,892
Number of nucleotides	191,715,599	404,520,160
Mean read length ± s.d. (bp)	249±151	397±152
**Reads Trimmed for Primers and Minimum Quality**		
Number of reads	483,938	900,490
Number of nucleotides	122,611,546	372,567,141
Mean read length ± s.d. (bp)	253±98	414±123
**Assembly**		
Number of contigs	5,062	16,846
Mean contig length ± s.d. (bp)	554±447	803±751
Median contig length (Lower/upper quartile)	464 (319/683)	614 (310/1041)
Contig N50 (N25/N75)	676 (462/1071)	1159 (697/1922)
Mean reads per contig	86.8	53.2
Median reads per contig (Lower/upper quartile)	12 (7/32)	13(7/34)
Mean read depth per contig	121.2	51.8
Median read depth per contig (Lower/upper quartile)	6.2 (4.3/13)	7.1 (4.7/13.1)
Contig GC Content	38.7%	35.7%
Number of singletons	46,353	53,449
Mean singleton length ± s.d. (bp)	243±103.5	380±136.6
Median singleton length (Lower/upper quartile)	251 (160/332)	417 (273/495)
Singleton GC Content	36.6%	33.9%

For both species, we tested three different assembly programs: Seqman NGen 3.1 (DNAStar Inc., USA), Trinity [39] and Newbler gsAssembler v 2.8 (Roche, Switzerland). The Newbler assembly was judged to provide the best assembly, due to longer reads and reduced redundancy (see Table S1 for more details). In addition, the Newbler assembly groups reads with polymorphisms and splice variations (see section below on isotigs and isogroups), therefore reducing redundancy without losing useful information. For these reasons, the Newbler assembly was used for the further analysis described below.

The mean and median contig length was longer for *O. nigriventris* than for *T. dichotomus* (Table 1). The adjusted median (N50) for *T. dichotomus* was 676 bp and 1,159 bp for *O. nigriventris* (Table 1), the longest contig for *T. dichotomus* was 9,123 bp, and 11,484 bp for *O. nigriventris* (a complete distribution of the contigs is given in Figure S1).

In both assemblies, a small number of contigs contained a high number of reads (e.g. >5,000 reads), thereby skewing the distribution of read number and coverage. For this reason, medians provide better metrics of the data (Table 1 and Table S2). The median number of reads per contig of 12 and 13 for the *T. dichotomus* and *O. nigriventris* respectively, with lower and upper quartiles of seven and 32 for *T. dichotomus*, and eight and 34 for *O. nigriventris* (Table 1). Approximately 20% of contigs contained more than 50 reads (*T. dichotomus*: 906; *O. nigriventris*: 2,861; Table S2).

### Isotigs and isogroups

In a second assembly step, contigs were grouped into “isotigs” and “isogroups”. During the assembly process, some reads (“split-reads”) form links between more than two different contigs. One possible reason is the presence of alternative splice variants of a gene, or other transcript differences (such as allelic variation, or unspliced UTRs). As part of the Newbler assembly method, contigs that can be connected by split-reads are grouped into “isogroups”. Within each isogroup, each possible different sequence is called an “isotig” (Figure S2). This step removes some of the redundancy present within a transcriptome assembly by grouping sequences that are likely from the same gene. The number of isogroups in an assembly can be seen as an approximation of the number of genes found, (although this can be affected by allelic variance and punctuated coverage of individual gene sequences).

In the *T. dichotomus* assembly, the contigs were condensed into 4,096 isogroups containing 4,507 isotigs between them, with an average of 1.3 contigs per isotig (Table 2). For the *O. nigriventris* assembly the contigs were condensed into 10,827 isogroups, containing 13,392 isotigs.

**Table 2:** Isogroup metrics.

	*T. dichotomus*	*O. nigriventris*
IsoGroups	4,096	10,827
Mean isotig count	1.1	1.3
Isotigs	4,507	13,392
Mean isotig length	690± 579	1326± 1164
Mean contigs per isotig	1.3	1.7

### Annotation and comparisons to *O. taurus* sequences

**Figure 2:**
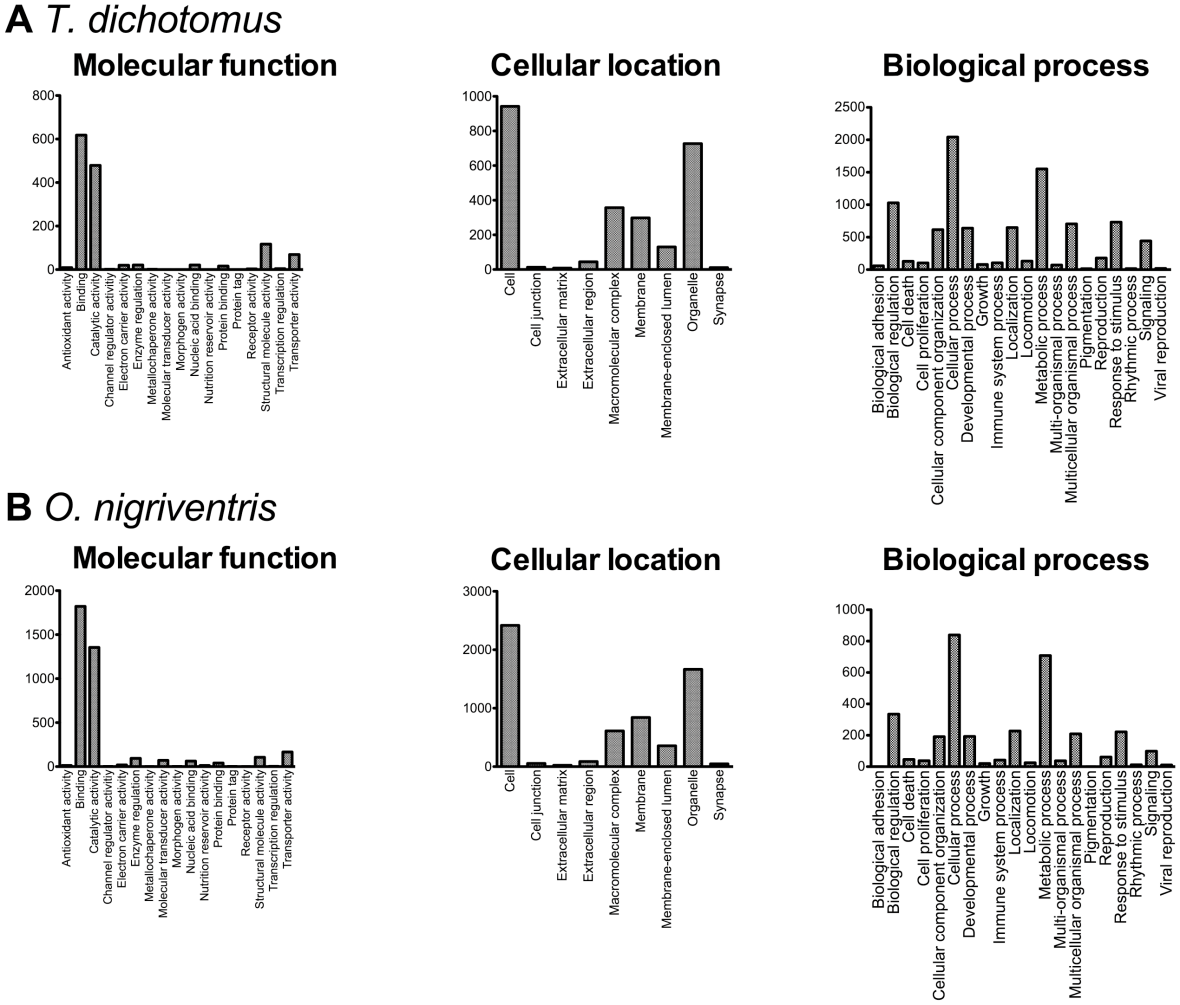
Distribution of level 2 GO Terms of BLAST hits from *T. dichotomus* (A) and *O. nigriventris* (B) assemblies. Note that a single entry in the UniProt database can be associated with more than one GO Term. A full list of all GO terms are given in Results File S1.

The assemblies generated were assessed by comparison to three separate databases. First, in order to provide a functional annotation, the assemblies were compared to a UniProt-Swissprot protein database [40]. Sequences from over 58% of *T. dichotomus* isogroups and over 65% of *O. nigriventris* isogroups returned positive hits (threshold  = *e* ^−5^). Over 2,173 unique GO terms were retrieved for the *T. dichotomus* assembly, and 4,141 unique GO terms were retrieved for the *O. nigriventris* assembly, which covered a wide range of functional categories (Fig. 2, Results File S1). The spread of GO terms across level 2 GO terms is typical of *de novo* transcriptome sequencing projects [41], [42].

**Figure 3:**
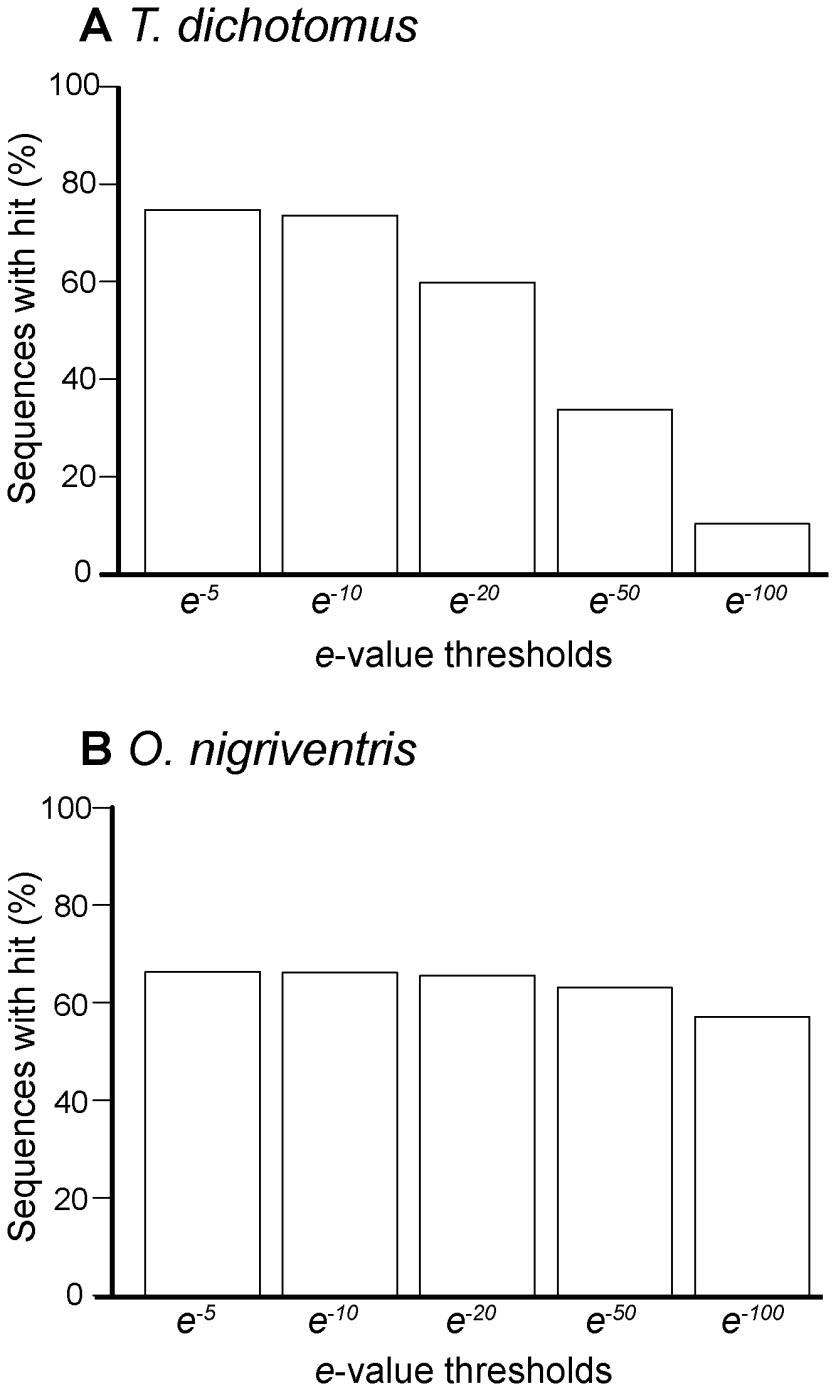
Comparisons of *T. dichotomus* (A) and *O. nigriventris* (B) assemblies against the *O. taurus* 454 transcriptome.

The primary aim of this project was gene discovery in the scarab beetle lineage, and therefore our second comparison was against the only scarab beetle species in the group with a transcriptome sequence, *O. taurus* [29] (Fig. 3). For *T. dichotomus*, at low thresholds (*e^−5^*) a large proportion of reads (∼75%) had matches in the published *O. taurus* transcriptome, but as the threshold increased (*e^−50^* and above), the proportion of reads with hits decreased to less than 50% (Fig. 3A). When the more closely related *O. nigriventris* sequences were compared to the published *O. taurus* transcriptome, approximately one third of sequences from the *O. nigriventris* assembly having no match, even at low *e*-value thresholds (Fig. 3B). This shows that we have generated a large amount of novel sequence data for the scarab beetle group and the *Onthophagus* genus. The *O. nigriventris* sequences without hits against the *O. taurus* transcriptome may arise due to the *O. taurus* transcriptome sampling a broad range of developmental stages and tissues, whereas here we focused on specific tissues and developmental stages. As a result, we sequenced at a greater depth in those tissues during developmental stages where high levels of gene expression would be expected.

The final comparison was against the core eukaryotic gene set (CEG; [43]), which represents 248 required genes in low paralogue number, expected to be seen in all eukaryotic species. This comparison provides another method for assessing the “completeness” of our transcriptomes. Both transcriptomes had a high number of hits against this database (threshold: *e* ^−5^; *T. dichotomus*: 232 hits (94%); *O. nigriventris*: 235 hits (95%)). This indicates that the transcriptomes contain a large portion of the transcripts expressed in the tissues we sampled.

### Single nucleotide polymorphism (SNP) detection

Even though a relatively small number of individuals were sampled for RNA (13 for *T. dichotomus*, 18 for *O. nigriventris*), we were able to detect single nucleotide polymorphisms (SNPs) within the assembled contigs that could be used in future studies. We made SNP calls at three levels of stringency: low (minor allele frequency (MAF) >15%, nucleotide depth >8); medium (MAF >20%, nucleotide depth >12); and, high (MAF >25%, depth >16). Furthermore, sequences had to be flanked by more than 50 bp of sequence on either side and potential SNP calls from long homopolymers were ignored since this is a common source of error in 454 sequencing [44]. For *T. dichotomus*, we retrieved between 2,222 (low stringency) and 759 (high stringency) potential SNPs across 739 and 270 contigs respectively (Table 3). For *O. nigriventris* between 17,453 (low stringency) and 3,911 (high stringency) potential SNPs were detected across between 4,002 and 1,424 contigs (Table 3). Full lists of potential SNPs are found in Results File S2.

**Table 3:** Summary of potential SNPs identified.

Species	Stringency	SNPs	Contigs	Transversions	Transitions
*T. dichotomus*	Low	2,222	739	624	1,598
	Medium	1,207	396	325	882
	High	759	270	217	542
*O. nigriventris*	Low	17,453	4,002	5,537	11,916
	Medium	7,246	2,206	2,203	5,043
	High	3,911	1,424	1,165	2,746

Low stringency  =  depth >8, minor allele frequency (MAF) >15%; medium stringency  =  depth >12, MAF>20%; high stringency: depth >12; MAF>25%.

### Molecular evolution: positive selection on horn-biased genes

Genes expressed in sexually-selected traits are under a range of selection pressures that are likely to affect their molecular evolution. This includes strong positive selection for genes under sexual selection, as well as relaxed purifying selection on genes that are not expressed in all individuals in every generation (e.g. horned vs. horn-less morphs).

We used the sequence data generated here for *T. dichotomus* and *O. nigriventris* as comparison sequences for a set of ∼4000 *O. taurus* that had been assayed for expression levels across different developing tissues using mirco-arrays [28]. From this point onwards, *O. taurus* because the focal study species, since this dataset has two properties that we investigated further: 1) It contained a small number of genes that were previously identified with horn-biased expression [27], [28]; 2) The degree of morph-biased expression (e.g. between horned males and horn-less males) has been calculated. In our first analysis, we tested whether genes with horn-biased expression were under positive selection. Using reciprocal tBLASTx searches (threshold  = *e* ^−5^) we compared the *O. taurus* sequences with *O. nigriventris* and *T. dichotomus* and subsequently made Ka/Ks estimations to test for signals of selection (Table 4). Positive selection (change in protein function) would be indicated by a higher proportion of non-synonymous changes (Ka) relative to synonymous changes (Ks), resulting in a Ka/Ks>1. Conversely, purifying selection for maintenance of the protein sequence would result in Ka/Ks<1, often close to zero.

**Table 4:** Descriptive statistics for Ka/Ks calculations.

	*T. dichotomus* vs. *O. taurus*	*O. nigriventris* vs. *O. taurus*
**All sequences**		
Number of comparisons	249	761
Mean alignment length (bp) (±standard deviation)	333.3(±170.0)	472.6(±222.2)
Mean Ka/Ks (±standard deviation)	0.2223 (±0.600)	0.2716 (±0.8522)
Median (lower/upper-quartile)	0.0741 (0.034/0.144)	0.064 (0.030/0.136)
Number of genes under positive selection (percentage of comparisons)	12 (4.8%)	50 (6.6%)
***O. taurus* head horn-biased genes**		
Number of comparisons	11	17
Mean Ka/Ks (±standard deviation)	1.170 (±1.387)	0.543 (±0.1.041)
Number of genes under positive selection (percentage of comparisons)	5 (45.5%)	3 (17.6%)

As expected, we found that the vast majority of genes are under purifying selection, with mean and median Ka/Ks values close to zero and low values for the upper quartile range (between 0.144 and 0.136; Table 4). A subset of genes tested (*T. dichotomus*: 12 out of 249; *O. nigriventris*: 50 out of 761), showed evidence of positive selection (Ka/Ks>1; Table 4). Within the *O. taurus* comparison with *T. dichotomus* it was possible to make Ka/Ks estimations from 11 homologue pairs involving genes that showed head horn-biased expression in *O. taurus* [28]. Five of these genes had signals of positive selection, and the mean Ka/Ks for all 11 genes was significantly greater than for the rest of the homologue pairs (Mann-Whitney, *W* = 2102, *p* = 0.00070).

For the comparison between *O. taurus* and *O. nigriventris*, the number of homologue pairs containing *O. taurus* horn-biased genes was 17, of which three showed evidence of positive selection. The mean Ka/Ks ratio was mildly significantly higher for genes with evidence of head horn-biased expression (Mann-Whitney, W = 8200, *p* = 0.036; Table 4). All of the annotated genes with head horn-biased expression in *O. taurus* that showed signals of positive selection are listed in Table 5.

**Table 5:** Best hits for BLASTx searches of *O. taurus* contigs with horn-biased expression and positive selection.

*O. taurus* contig	*T.dichotomus* homologue	*O. nigriventris* homologue	NCBI non-redundant database: Name; species; and accession number
	Sequence name	Ka/Ks	Sequence name	Ka/Ks	
contig36648_c923	isotig01228	3.81	isotig07348	3.66	PREDICTED: similar to cuticular protein 1, RR-2 family; Tribolium castaneum; XP_975671
contig00068_a5	isotig01980	3.26	isotig01276	NA	No hit
contig12109_a301	isotig01570	2.28	isotig13531	2.28	Full = Cuticle protein LPCP-23; Tenebrio molitor; Q94804
contig13345_b878	isotig01176	1.48	isotig12229	1.88	PREDICTED: similar to cuticular protein 1, RR-2 family; Tribolium castaneum; XP_975669
OtL002-B04	isotig00660	1.28	isotig10739	0	No hit

### Molecular evolution: developmental decoupling on morph-biased genes

Our second analysis assessed the effect of developmental decoupling on gene evolution [28], [36], [37]. The theory of developmental decoupling predicts that genes with increased morph-biased expression (e. g. horned vs. horn-less morphs) should be subject to relaxed purifying selection [28], [36], [37] since they will only be expressed in a subset of the population in each generation. In the study from which we obtained the sequences [28], comparisons had been made to estimate selection by examining sequence divergence between predicted *O. taurus* protein sequences and *T. castaneum* protein sequences. Consistent with the theory of developmental decoupling, their results suggest that increased morph-biased expression was correlated with increased rates of amino acid change [28]. These results were provided a novel insight into the effects of developmental decoupling in scarab beetles, but were limited by the use of two long-diverged species (approximately 250 MYr [45]), therefore potentially saturating nucleotide changes and reducing the ability to detect different levels and types of selection. *O. nigriventris* is a congener of *O. taurus* (the *Onthophagus* genus radiated about 20–33 million years ago [6], [46], [47]), and *T. dichotomus* is separated from *O. taurus* and *O. nigriventris* by about 100 Myr [18], . We therefore utilised our comparisons to build upon the previous result and test the relationship between morph-biased expression and purifying selection more explicitly, using nucleotide data.

**Figure 4:**
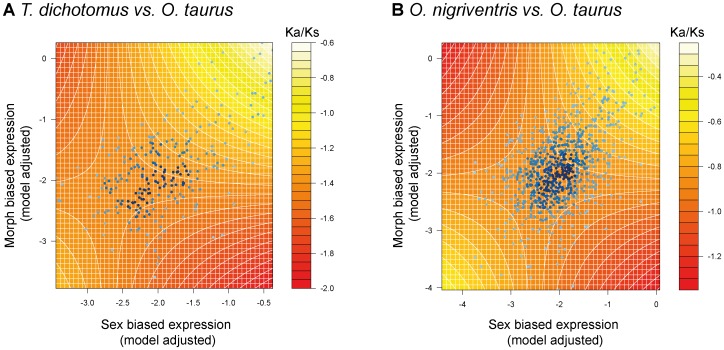
Effects of morph-biased and sex-biased expression on Ka/Ks in horned beetles. Contour plots of the effects of morph-biased expression and sex-biased expression on Ka/Ks in *O. taurus* vs *O. nigriventris* (A) and *O. taurus* vs *T. dichotomus* (B) comparisons. The contours are predicted from the general linear models given in Table 6. Super-imposed (blue dots) are the model adjusted values for sex-biased expression and morph-biased expression. These represent the averaged differences in gene expression between sexes and between horned- and horn-less males. These were previously calculated in [28]. Sex-biased expression, morph-biased expression and Ka/Ks have been log-transformed.

We recreated the general linear model (GLM) from Snell-Rood *et al.* [28], with the addition of sequence alignment length, along with the interaction term of morph-biased and sex-biased expression (see methods for details of the model). For the comparison between *T. dichotomus* and *O. taurus* (Table 6), sex-biased expression, morph-biased expression, and their interaction term, had positive and significant relationships with Ka/Ks ratio, although the variation accounted for by these terms is modest (partial r^2^ = 0.019–0.039; Fig. 4A), suggesting that other evolutionary forces are contributing substantially to variation in Ka/Ks (Fig. 4A). Alignment length showed a stronger, but negative, correlation with Ka/Ks ratio (partial r^2^ = 0.047), which is probably due to an artefact of the protein prediction method used (see discussion and methods).

**Table 6:** Summaries of general linear models testing effects on Ka/Ks ratio.

Effect term	Parameter estimate ± SE	Partial *r^2^*	*t*-value
***T. dichotomus vsO. taurus***			
Overall expression	0.0263±0.024	0.005	1.104
Alignment length	−0.0007±0.0002	0.047	−3.443***
Sequence length	0.0001±0.0001	0.003	0.798
Nos. tissues/arrays	0.0434±0.034	0.007	1.266
Sex-biased expression	0.315±0.133	0.023	2.368*
Morph-biased expression	0.384±0.122	0.039	3.147**
Morph-biased ⋅ Sex-biased expression	0.144	0.019	2.163*
***O. nigriventris vs O. taurus***			
Overall expression	−0.013±0.016	0.0010	−0.802
Alignment length	−0.0008±0.0001	0.0752	−7.829***
Sequence length	−0.0002±0.00010	0.0057	2.087*
Nos. tissues/arrays	0.0192±0.0208	0.0011	0.355
Sex-biased expression	0.201±0.0838	0.0076	2.400*
Morph-biased expression	0.230±0.0817	0.0104	2.812**
Morph-biased ⋅ Sex-biased expression	0.0917±0.040	0.0066	2.243*

Shown above are parameter estimates for the general linear model testing the effects against Ka/Ks for between *O. taurus* and *O. nigriventris*, and for *O. taurus* against *T. dichotomus*. *Log (Ka/Ks)* is the dependent variable. The effect terms*: overall expression*, *sequence length*, *alignment length*, *sex-biased expression* and *morph-biased expression* have all undergone log-transformations. For the model comparing *T. dichotomus* with *O. nigriventris* the interaction term of *morph-biased expression* and *sex-biased expression* was removed. Full model adjusted *r^2^* for *O. nigriventris* vs. *O. taurus* was 0.105, and 0.150 for the comparison between *O. taurus* and *T. dichotomus*. All numbers are quoted to three decimal places or two significant figures when required. Degrees of freedom for the t-tests were 753 (n = 761) for the *O. nigriventris* vs. *O. taurus* comparison, and 241 (n = 249) for the *T. dichotomus* vs. *O. taurus* comparison. Asterisks denote significance of the t-value as follows: * p<0.05, ** p<0.01, *** p<0.001.

For the *O. taurus* comparison with *O. nigriventris* (Table 6), as with the previous comparison, both morph-biased and sex-biased expression, along with their interaction, were observed to have very small, but positive and significant relationships with Ka/Ks, (partial *r* ^2^ = 0.0066–0.0104) (Fig. 4B). Alignment length as well as sequence length had negative relationships with Ka/Ks, with alignment length accounting for slightly more variation (partial *r* ^2^ = 0.0752).

We found a positive relationship between both sex-biased and morph-biased gene expression and Ka/Ks ratio in both the *O. taurus* and *T. dichotomus* comparison, and in the *O. taurus* and *O. nigriventris* comparison. These results are consistent with relaxed purifying selection associated with morph-biased expression.

In summary, our data provide evidence for two mechanisms thought to be critical to rapid evolution of extreme sexually selected structures: recent genetic change resulting from directional selection on sexually selected traits in a few genes, along with a more broadly distributed accumulation of genetic variation resulting from relaxed purifying selection in genes that have sex- or morph-specific facultative expression of ornaments and weapons.

## Discussion

Horned beetles are an excellent emerging model system for studies of the genetic and physiological basis of sexual selection and phenotypic plasticity. Transcriptomes of two horned beetle species, combined with the existing transcriptome of *Onthophagus taurus* [29] and the partial methylome of *O. gazella* [48], provide a resource for advancing research using horned beetles and their exaggerated sexually selected traits. More specifically, our analyses have begun to identify genes under positive selection in this lineage, possibly due to their involvement in horn development, along with evidence for historical selection pressures on genes involved in horn development and differentiation of morphs (horned vs. horn-less morphs).

### Transcriptome assembly and annotation

Both transcriptomes returned a high number of unique sequences and a wide range of functions in their GO annotations. The number of reads, contigs and isogroups, were markedly different between the two species sequenced (Table 1). These differences can be attributed to two main factors. First, the focal species in this study are very different in overall size (Fig. 1) and, as a result, our sampling methods differed. Due to the large size of *T. dichotomus* we could dissect and obtain RNA from specific developing tissues. For *O. nigriventris*, this was not possible and we used whole body samples at developmentally relevant stages. This may explain the greater number of *O. nigriventris* contigs (Table 1 & 2) because of the higher number of genes expressed in whole bodies, and the subsequent increased number of alternatively spliced isotigs [49].

The second factor that influenced the total number of reads was the starting material for the synthesis of the sequencing library, as well as the method of preparation of the sequencing library, which reflects a trade-off inherent in our sampling approaches. In general, it is optimal to make a 454-sequencing library directly from mRNA [50]. However, sampling individual tissues for *T. dichotomus* yielded an insufficient amount of total RNA for mRNA enrichment, therefore cDNA was synthesised and amplified. The whole-body sampling of *O. nigriventris* generated enough total RNA for mRNA-enrichment prior to library preparation. This may have caused the relative reduction in length and number of the reads in the *T. dichotomus* library (Table 1). Despite the methodological limitations, both transcriptomes provide a rich list of genes expressed during imaginal tissue development for both species.

### SNP Discovery

SNP detection using a range of threshold levels that avoid sequencing and assembly errors, identified a large number of variable sites in both *O. nigriventris* and *T. dichotomus*. These provide a new resource for mapping genes linked to variation in trait size in selection experiments, as well as for use in population genetic studies [51]. For example, between naturally occurring populations of *T. dichotomus* there is a high degree of variation in the allometry of horn-size and body size based on geography and reproductive isolation; populations from the main island of Japan (Honshu island; *T.d. septentrionalis*) have heritable differences in head horn length when compared to populations from Okinawa island (*T.d. takarai*,) [22]. For the dung beetle *O. nigriventris*, populations were introduced in the 1960s to both Eastern Australia and Hawaii. These populations now differ strikingly in their thoracic horn length, comparable in magnitude to previously published differences between Australian and North Carolina populations of *O. taurus* [23]. These SNP sites can be analysed for allele frequencies within and across populations, thereby identifying loci associated with horn allometry variation. In addition, as Helyar *et al.* [51] point out in their recent review on the use of SNPs as markers for non-model organisms in studies of population structure, we are on the verge of being able to use genomic tools to gain a general understanding of the importance of neutral and adaptive processes in wild populations. Genomic studies on the developmental biology of beetle horns promise to contribute substantially to this.

### Selection on weapons in horned beetles

Exaggerated ornaments and weapons of sexual selection are thought to experience unusual patterns of selection relative to other body traits, for two reasons. First, genes that contribute to increased horn size will be under strong positive selection due to the fitness benefits they confer (e.g. increased mating success) [52], [53]. Second, because these traits are dimorphically expressed between sexes and, in some instances, dimorphically expressed within sexes (as in scarab beetles such as *O. nigriventris*), the genes involved in their development will be expressed within the population less frequently and therefore the effects of purifying selection are predicted to be lower.

Here we provide evidence for positive selection in genes with horn-biased expression, along with relaxed purifying selection in genes that show developmental decoupling. Both directional positive selection and relaxed purifying selection may be important factors in generating phenotypic diversity, such as seen amongst scarab beetles, since they provide mechanisms for changes in gene sequence, structure and, therefore, function. Indeed, in social insects, it is proposed that relaxed purifying selection is key to generating phenotypic diversity [38].

In the aforementioned study of phenotypic plasticity in social insects [38], although relaxed purifying selection on developmentally decoupled genes was detected, there was little evidence for positively selected genes (three out of 1000). In our beetle study the percentage of positively selected genes was an order of magnitude greater (4.8%–6.6%; Table 4), which might be expected since the roles of the beetle morphs are directly related to reproductive strategies and success, and therefore some genes have the potential to be under strong positive sexual selection.

The function of the genes with evidence of head-horn enriched expression along with evidence of positive selection is unclear, and will require further annotation. When compared to available databases, only three out of the five genes gave significant hits, and two of those were against predicted proteins from genome sequencing projects (Table 5). For the three genes where significant hits were retrieved, they were implicated in being involved in cuticle development. The beetle horns are hollow cuticular structures, so it is expected that cuticle related genes maybe up-regulated. The positive selection on these particular genes may indicate that horn growth requires an alteration in cutcile structure for exaggerated growth and/or for re-enforcement of a structure used in fighting.

The sequence comparisons used here may in fact underestimate the degree of selection because, in order to maximize accuracy, protein predictions were based on over-lapping tBLASTx search results and are therefore restricted to comparisons of the most strongly conserved regions; this may explain the negative relationship observed between alignment length and Ka/Ks ratio. Using global alignment strategies, or BLAST searches with lower threshold values for extension, may increase the level of sequence variation detected and increase Ka/Ks estimates. Upon completion and annotation of the *O. taurus* genome sequence [54], such approaches will be achievable, but they are currently computationally problematic and will sacrifice accuracy of the protein prediction and subsequent Ka/Ks estimations.

In addition to the methodological considerations described above, there are likely to be other factors that limit the general linear model described here (Table 6). Genes evolve and nucleotide sequences change due to a variety of factors, including various agents of selection, as well as stochastic processes such as population bottlenecks and genetic drift. Clearly it is unlikely that we have captured all the factors, and this is evidenced by the low partial *r^2^*-values observed here (Table 6). However, low coefficients of determination are typical for studies of molecular evolution (e.g. [55], [56]), so this is not unexpected. We have included as many factors as possible that are known to influence gene evolution (e.g. overall expression level [55]) and there are clear signals in our models demonstrating the effects of morph-biased expression on gene evolution.

From the point of view of refinement of the general linear model used here (Table 6) and for future directions of research, there are other potential factors affecting gene evolution such as non-coding DNA and gene interactions. In our study, we examined only coding regions of the genes, whereas the evolution of non-coding cis-regulatory regions that regulate gene expression are expected to play an important role in trait evolution [57]. Furthermore, genes do not evolve independently, indeed the origin of novel traits, such as the scarab beetle horn, is proposed to involve the co-option of pre-existing gene networks [58], [59]. Such interactions between genes may exaggerate any effects of selection, in that change in one gene may cause selection on interacting genes. This should only be a significant effect under strong positive selection for protein sequence and structure, rather than under the relaxed purifying selection predominantly observed in this study. As more data become available, such as the up-coming sequence of the *O. taurus* genome [54], it will be possible to build on the model used here and investigate the importance and role of cis-regulatory DNA, non-coding regions, and gene networks in the evolution of beetle horns.

## Conclusions

Scarab beetles are an emerging model system for understanding the growth of exaggerated traits. Here we have sequenced, assembled, and annotated transcriptomes for two scarab beetles, *Trypoxylus dichotomus* and *Onthophagus nigriventris*. These data will provide an important resource for future studies in beetle horn development as well as understanding the evolution and development of exaggerated sexually selected traits.

We also used the data to test for signals of selection in genes showing morph-biased expression in scarab beetles; these results provided strong evidence that horn-biased genes are affected by strong positive selection due to sexual selection, as well as relaxed purifying selection due to phenotypic plasticity of the trait.

## Methods

### Beetle rearing

*Trypoxylus dichotomus* individuals were reared at the Fort Missoula Research Station at the University of Montana following methods described in [60]. To briefly summarise, adults were paired in gallon jars and fed with sliced apples. After seven days females were isolated and, after a further seven days, eggs were collected. Eggs were hatched in plastic cups with 100% mulched maple leaves. Upon hatching, larvae were reared in 9 oz glass jars containing 25% mulch and fermented hardwood sawdust and monitored for development.

*Onthophagus nigriventris* individuals were reared from individuals collected at the Kahua Ranch, Kamuela, Hawaii 96743 (Lat: 20.116812, Long: −155.788450) in late May 2010. Adults were bred in 150 cm^2^ cylindrical plastic containers containing 600 ml sterilised sand and 200 ml cow dung at 26°C on a 16∶8 light∶dark cycle. Two females and one male were kept in each container with sand and cow dung being changed every two weeks. Any brood balls [61] containing eggs were removed and the developing larvae within were monitored.

### Dissection and RNA extraction

For both species, we targeted developing tissues covering the main period of horn growth for sequencing (Fig. 1E), although the exact tissues surveyed and the sample preparation method differed between *T. dichotomus* and *O. nigriventris*, with extraction methods being tailored for specific tissues. In *T. dichotomus*, RNA was collected from the following 11 tissues: head horn, thoracic horn, brain, genitals, fat body and forelegs from pre-pupal males (Fig. 1E); head, thorax and genitals from late 3^rd^ instar males (Fig. 1E); and head and thorax from late 3^rd^ instar females (Fig. 1E). Samples were taken from a total of seven individuals. Animals were anaesthetised using CO_2_, tissues removed and transferred directly to RNAlater® (Applied Biosystems, USA), and stored at −80°C until extraction. For most tissues, following homogenisation using a handheld electric tissue tearer, RNA was extracted using the Qiagen RNeasy® RNA extraction kit (Qiagen, USA) following the manufacturer's protocol. For larval head tissues, dissected tissue was directly transferred to, and homogenised in, TRiZOL® (Invitrogen, USA) and then left overnight at −80°C, before extraction following manufacturer's protocol. RNA was dissolved in 40 µl nuclease free water. For fat-body tissues both the TRiZOL® and Qiagen RNeasy® tissue methods were used sequentially to provide RNA of sufficient quality and quantity.

For *O. nigriventris*, four RNA libraries were made from whole individuals of both sexes during critical periods of horn growth: the first library contained male individuals from the late pre-pupal period; the second contained female individuals from the same time point; the third library contained male individuals from the 3^rd^ instar and early pre-pupa stages; and the final library contained female individuals from the 3^rd^ instar and early pre-pupa as well (Fig. 1E). All digestive tracts were removed and individuals were homogenised in TRiZOL® (Invitrogen, USA) and stored overnight at −80°C before extraction following manufacturer's instructions. RNA was dissolved in 40 µl nuclease-free water. RNA was extracted from a total of 18 individuals.

All RNA extractions were then treated with Ambion® DNA-*free*™ DNase, before assaying quantity, with a NanoDrop (Thermo Scientific, USA), and quality, with a BioAnalyzer 2100 (Agilent Inc., USA). Briefly, from each RNA extraction, two aliquots were taken. One was left at 37°C for two hours and the other at 4°C for two hours. Both aliquots were then run on an Agilent RNA nano-chip (Agilent Inc., USA). The resulting electropherograms were compared and if either showed signs of degradation in the ribosomal peaks, or in general, then the sample was discarded. It should be note that an RIN value system [62] is not applicable for beetle species as, like many other insect species, the 28S ribosomal peak collapses and merges with the 18S ribosomal peak upon heating [63].

### cDNA library preparation and sequencing

For the 11 *T. dichotomus* libraries, full-length enriched cDNA was synthesised and amplified from 1 µg of total RNA using the SMARTer™ PCR cDNA synthesis kit (BD Clontech, USA). Purification of cDNA was carried out using the Agencourt® AMPure® XP (Beckman Coulter, USA) and checked for quantity and quality on a NanoDrop (Thermo Scientific, USA) and a BioAnalyzer 2100 (Agilent Inc., USA). The resulting electropherograms were checked for presence of any peaks that would indicate PCR bias in the cDNA amplification step. If such peaks were present then the library was synthesised again.

For the four *O. nigriventris* libraries, total RNA was enriched for mRNA using Oligotex® mRNA purification kit (Qiagen Inc., USA). The protocol was run twice to increase the proportion of mRNA present. Preparations were visualised using a BioAnalyzer (Agilent Inc., USA) to check that mRNA had been enriched (e.g. reduction in the visible ribosomal RNA peaks), and were quantified on a NanoDrop (ThermoScientific, USA).

Aliquots of *T. dichotomus* cDNA and *O. nigriventris* mRNA were taken to the Washington State University Molecular Biology Core Laboratory for library preparation, and sequenced using the 454 GS FLX Titanium Series (Roche, USA) following manufacturer's protocols. Briefly, *T. dichotomus* cDNA was fragmented by nebulisation and used to create the libraries. For *O. nigriventris*, mRNA-enriched RNA was fragmented by ZnCl_2_ and heat prior to cDNA synthesis. Different molecular identifiers were ligated to each sample during library construction so that individual reads could be traced to their tissue of origin once assembled. Libraries were titrated to achieve 8–10% enrichment and the sequencing beads for each library were made in separate reactions. Sample balance was achieved by loading the picotiter plate with an equal number of enriched beads, determined by coulter counter, for each library. Reads were deposited to the NCBI Sequence Read Archive (http://trace.ncbi.nlm.nih.gov/Traces/sra/), accession numbers: SRR1049790 to SRR1049795, and SRR1049815 to SRR1049821 for *T. dichotomus*; SRR1049522 SRR1049767, SRR1049768 and SRR1049789 for *O. nigriventris*.

### Transcriptome assembly and annotation

Pre-assembly processing was performed using PipeMeta version 0.44, a freely available script designed to help run a *de novo* transcriptome annotation pipeline [49], [64]. SMARTer primer sequences were trimmed from the reads and the individual reads filtered for quality (minimum average quality score  = 20, maximum degenerate base [N] calls  = 1, minimum/maximum length  =  avg. length ±2×SD). A small number of useable reads (37,706) recovered from a previous 454 sequencing run for *T. dichotomus* that had been contaminated by primer-dimers were also included in the assembly. These were originally sourced from pooled RNA from the legs, wings, genitals and head horns across nine males in the pre-pupal stage. They are referred to as “library 12”.

Three different assembly approaches were tested for both species: 1) Seqman NGen 3.1 (DNAStar, Inc., USA; match size  = 21, match spacing  = 75, min. match pair  = 90, use repeat handing  =  false, remove small contigs  =  true, min. seqs  = 2, min. length  = 50; 2) The Broad Institute's Trinity pipeline [39] using default parameters; 3) Newbler gsAssembler v 2.8 (Roche, Switzerland; seed step  = 12, seed length  = 16, seed count  = 1, minimum overlap length  = 40, minimum overlap identity  = 90%, alignment identity score  = 2, alignment difference score  = −3). Assemblies using Newbler were judged to be the best. The assemblies are available in Sequence Data S1 and S2 and have been deposited at DDBJ/EMBL/GenBank (see “Data accessibility” for accession numbers).

In order to assign functional annotation to the assemblies, they were compared to using BLASTx searches (threshold  = *e* ^−5^) to a UniProt protein database (downloaded April 2013) [40]. In order to avoid over-representation of sequences where multiple isotigs are present in the same isogroup, only the isotig with the lowest *e*-value was selected for further functional analysis. Mapping of GO terms and subsequent filtering was performed in BLAST2GO [65].

A second comparison was carried out against a modified version of the *O. taurus* transcriptome [29] (see below) using BLASTn at a range of *e*-value thresholds. A final comparison was performed against the Core Eukaryotic Dataset [43] (BLASTx, threshold  = *e* ^−5^), which is a set of 248 essential genes present in a wide range of eukaryotes in low paralogue number.

### SNP identification

Single nucleotide polymorphisms (SNPs) detection was carried out based on a method adapted from [66]. Raw filtered reads were mapped back to the 454AllContigs.fna file from the Newbler assembler output, using Roche's gsReference Mapper (v2.8) (Seed step  = 12, seed length  = 16, seed count 1, Hit-per-seed limit  = 70; minimum overlap length  = 40, minimum overlap identity  = 90, alignment ID score  = 2, alignment differences score  = −3). Custom python scripts were written that filtered the resulting 454AllDiffs.txt file for single nucleotide differences. We performed this at three threshold levels: Low stringency SNPs had a nucleotide depth greater than eight, and a minority allele frequency (MAF) greater than 15%; medium stringency SNPs had a nucleotide depth greater than 12, and a MAF greater than 20%; high stringency SNPs had a nucleotide depth greater than 15 and a MAF greater than 25%. In additional, all putative SNPs had to be flanked by at least 50 bp of sequence, and were not part of a single nucleotide repeat longer than two nucleotides.

### Comparisons with *Onthophagus taurus* sequences

To enable comparisons of the transcriptome sequences against *Onthophagus taurus*, two *O. taurus* sequence datasets were merged, a Sanger generated EST library [27] and a 454 transcriptome [29]. This was necessary as the Sanger generated ESTs from Kijimoto *et al.* contained biologically relevant information, such as tissue-specific expression levels (including head horn), and levels of sex-biased and morph-biased expression [28], but had not been assembled in the *O. taurus* 454 dataset. Having an increased number of sequences in the database increased the strength of reciprocal BLAST searches [67] to identify true homologues between the Sanger generated ESTs and the *T. dichotomus* and *O. nigriventris* datasets. In addition, since the Sanger and 454 transcriptomes were from two different sequencing runs, in order to reduce redundancy, the two transcriptome datasets were merged as described below.

A reciprocal BLASTn search (threshold  = *e^−5^*) was performed between the *O. taurus* Sanger generated ESTs and the *O. taurus* 454 transcriptome. For positive hits were assembly together using CodonCode Aligner (CodonCode Corporation, USA). Several assemblies were trialed using different parameters, then visually inspected to check the assemblies were correct, and parameters of 90% identity and maximum gap extension penalties were judged to be optimal. *O. taurus* sequences assembled under these conditions were used in place of the original sequence in the 454 database. If the EST sequences did not assemble then they were added to the 454 database without modification (the modified assembly is presented in Sequence Data S3).

### Molecular evolution

Reciprocal tBLASTx searches were carried out between the *T. dichotomus* transcriptome, the *O. nigriventris* transcriptome and the modified dataset of over 1500 *O. taurus* ESTs combined with 454 sequences (see above; [28]). DNA sequences were trimmed to the overlapping regions from the tBLASTx alignment and then translated in the reading frame of the tBLASTx search using a custom Python script, before being aligned using MAFFT [68]. Stop codons and nonsense codons were removed. DNA sequences were then aligned to the predicted protein sequence using PAL2NAL [69] and Ka/Ks calculations performed using the KaKs Calculator tool [70], implementing the Nei and Gojobori method for Ka/Ks estimation [71]. We used alignments of less than 150 bp were discarded to ensure sufficient distances for reliable Ka/Ks calculations (e.g. 50 or more codons). Differences in Ka/Ks ratio between homologues containing genes with horn-biased expression and the homologues not containing genes with horn-biased expression patterns, were tested using Mann-Whitney test carried out in R version 2.15.1 [72].

Values of morph-specific expression bias, sex-specific expression bias, number of tissues expressed and overall (average) expression had been calculated previously for the *O. taurus* ESTs [28]. During the assembly of the Sanger EST sequences with the 454 database, occasionally more than one Sanger EST was assembled within one 454 sequence. When this occurred all expression values for the sequences involved were then averaged (see Materials and Methods S1). Sequence was given as the length of the new contig. The following general linear model was constructed (based on the model in Snell-Rood *et al.* [28]): 
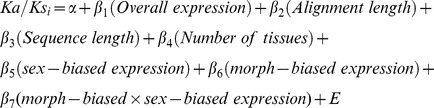
(*α* =  intercept, *E* =  error). Estimations of Ka/Ks, sex-specific expression and morph-biased expression were all log-transformed. The model was constructed and tested using the lm() function and custom functions for computing the partial *r^2^* [73] in R version 2.15.1 [72]. We also examined the potential for non-linear influences of sex and morph-biased expression with explicit power and orthogonal polynomials. In no case did the inclusion of quadratic terms improve model fit, or were individual quadratic terms having biologically or statistically significant effects. We also tested for any effects of colinearity between the variables, and no significant effects were found. Code for all linear model fits, including model diagnostics are available in Materials and Methods S2 and on DRYAD (doi:10.5061/dryad.n10j0). The significance of each parameter estimate was estimated using a t-test.

## Data Accessibility

Raw sequence reads were deposited to the NCBI Sequence Read Archive (http://trace.ncbi.nlm.nih.gov/Traces/sra/), accession numbers: SRR1049790 to SRR1049795, and SRR1049815 to SRR1049821 for T. dichotomus; SRR1049522 SRR1049767, SRR1049768 and SRR1049789 for O. nigriventris.

The assemblies have been deposited at DDBJ/EMBL/GenBank under the accessions GAQV00000000 (T. dichotomus) and GAQW00000000 (O. nigriventris). The versions described in this paper are the first versions, GAQV01000000 (T. dichotomus) and GAQW01000000 (O. nigriventris).

All sequences, raw data, and codes used in the manuscript are available at DRYAD, doi:10.5061/dryad.n10j0.

## Supporting Information

Figure S1 **Cumulative frequency curves for contig length from assemblies of reads from 454 sequencing for *T. dichotomus* and *O. nigriventris*.**
(DOC)

Figure S2 **Isotig/isogroup explanation.**
(DOC)

Materials and Methods S1 **Adjusted morph-biased expression values for** ***O. taurus*** **.**
(XLS)

Materials and Methods S2 **R-code for molecular evolution analysis.**
(R) Click here for additional data file.

Results File S1 **Summary of all GO terms for *T. dichotomus* and *O. nigriventris* assemblies.**
(XLSX)

Results File S2 **Summary of all potential SNP calls from** ***T. dichotomus*** **and** ***O. nigriventris*** **.**
(XLSX)

Sequence Data S1 ***Trypoxylus dichotomus* assembly.**
(FASTA)

Sequence Data S2 ***Onthophagus nigriventris* assembly.**
(FASTA)

Sequence Data S3 **Modified *Onthophagus taurus* 454 transcriptome.**
(FASTA)

Table S1 **Comparisons between assemblies.**
(DOCX)

Table S2 **Cumulative frequency of read counts per contig from 454 sequencing for** ***T. dichotomus*** **and** ***O. nigriventris*** **.**
(DOC)
