# Supplementary material for: Insights into the Development and Evolution of Exaggerated Traits Using De Novo Transcriptomes of Two Species of Horned Scarab Beetles
Source: PLoS One. 2014 Feb 20;9(2):e88364. doi: 10.1371/journal.pone.0088364 (PMC3930525; doi:10.1371/journal.pone.0088364)
Supplement: Figure S1 — Cumulative frequency curves for contig length from assemblies of reads from 454 sequencing for T. dichotomus and O. nigriventris. (DOC) [file pone.0088364.s001.doc]

Figure S1A: Relative cumulative frequency of contig length in the *T. dichotomus* assembly

Figure S1B: Relative cumulative frequency of contig length in the *O. nigriventris* assembly
