# Supplementary material for: Insights into the Development and Evolution of Exaggerated Traits Using De Novo Transcriptomes of Two Species of Horned Scarab Beetles
Source: PLoS One. 2014 Feb 20;9(2):e88364. doi: 10.1371/journal.pone.0088364 (PMC3930525; doi:10.1371/journal.pone.0088364)
Supplement: Figure S2 — Isotig/isogroup explanation. (DOC) [file pone.0088364.s002.doc]

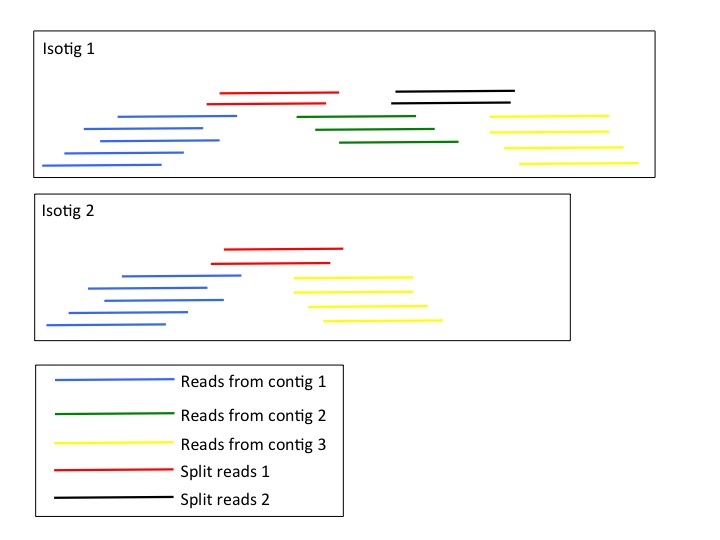


Figure S2: Schematic representation of two isotigs from an isogroup. Split reads 1 can connect reads from contig 1 to reads from both contig 2 and contig 3. Split reads 2 can only connect reads from contig 2 and contig 3. As a result, Newbler creates two isotigs containing both possible arrangements of reads. Also see: Choi *et al* 2010 BMC Genomics **11**: 703
