# Supplementary material for: Insights into the Development and Evolution of Exaggerated Traits Using De Novo Transcriptomes of Two Species of Horned Scarab Beetles
Source: PLoS One. 2014 Feb 20;9(2):e88364. doi: 10.1371/journal.pone.0088364 (PMC3930525; doi:10.1371/journal.pone.0088364)
Supplement: Table S1 — Comparisons between assemblies. (DOCX) [file pone.0088364.s010.docx]

| **Assembly** | **NGen** | **Trinity** | **Newbler** |
| --- | --- | --- | --- |
| Number of contigs | 17,679 | 5,064 | 5,062 |
| Mean contig length ± s.d. (bp) | 471 ± 298 | 468 ± 224 | 554 ± 447 |
| Median contig length (Lower/upper quartile) | 390 (311/553) | 393 (341/507) | 464 (319/683) |
| Contig N50 (N25/N75) | 513 (372/780) | 445 (362/647) | 676 (1071/462) |
| Mean reads per contig | 24.85 | 54.4 | 86.8 |
| Median reads per contig (Lower/upper quartile) | 3 (2/6) | 12 (6/23.25) | 12 (7/32) |
| Mean read depth per contig | 9.42 | 34.99 | 121.2 |
| Median read depth per contig (Lower/upper quartile) | 2 (1.6/3.4) | 7.8 (4.8/14.8) | 6.2 (4.3/13) |
| Number of singletons | 43,343 | 86,970 | 46,353 |

Table S1A: *T.dichotomus* assembly metrics

Table S1B: *O. nigriventris* assembly metrics

| Assembly | **NGen 3.1** | **Trinity** | **Newbler** |
| --- | --- | --- | --- |
| Number of contigs | 63,270 | 48,777 | 16,846 |
| Mean contig length ± s.d. (bp) | 670 ± 414 | 544 ± 403.5 | 803 ± 751 |
| Median contig length (Lower/upper quartile) | 561 (469/797) | 433 (301/624) | 614 (310/1041) |
| Contig N50 (N25/N75) | 734 (531/1151) | 606 (411/1026) | 1159 (697/1922) |
| Mean reads per contig | 13.65 | 13.07 | 53.2 |
| Median reads per contig (Lower/upper quartile) | 3 (2/7) | 7 (4/12) | 13 (7/34) |
| Mean read depth per contig | 5.61 | 8.95 | 51.8 |
| Median read depth per contig (Lower/upper quartile) | 2.5 (1.8/4.3) | 4.9 (3.0/8.1) | 7.1 (4.7/13.1) |
| Number of singletons | 37,029 | 23,483 | 53,449 |
