# Supplementary material for: Insights into the Development and Evolution of Exaggerated Traits Using De Novo Transcriptomes of Two Species of Horned Scarab Beetles
Source: PLoS One. 2014 Feb 20;9(2):e88364. doi: 10.1371/journal.pone.0088364 (PMC3930525; doi:10.1371/journal.pone.0088364)
Supplement: Table S2 — Cumulative frequency of read counts per contig from 454 sequencing for T. dichotomus and O. nigriventris . (DOC) [file pone.0088364.s011.doc]

Table S2: Cumulative frequency of read counts per contig

| **Number of reads** | **2** | **≤3** | **≤4** | **≤5** | **≤6** | **≤7** | **≤8** | **≤9** | **≤10** |
| --- | --- | --- | --- | --- | --- | --- | --- | --- | --- |
| Number of *T. dichtomus*  contigs (%) | 78  (1.6) | 231  (4.7) | 305  (6.2) | 363  (7.4) | 914  (18.6) | 1346  (27.5) | 1688  (80.6) | 1940  (39.6) | 2170  (44.3) |
| Number of  *O. nigriventris* contigs (%) | 1067  (6.8) | 1614  (10.3) | 2023  (12.9) | 2380  (15.2) | 3544  (22.6) | 4545  (29.0) | 5381  (34.3) | 6108  (39.0) | 6716  (42.8) |
|  |  |  |  |  |  |  |  |  |  |
| **Number of reads** | **≤15** | **≤20** | **≤30** | **≤40** | **≤50** | **≤100** | **≤250** | **≤500** | **≤1000** |
| Number of *T. dichtomus*  contigs (%) | 2847  (58.1) | 3211  (65.6) | 3644  (74.4) | 3856  (78.7) | 3992  (78.7) | 4348  (88.8) | 4611  (94.1) | 4737  (96.7) | 4834  (98.7) |
| Number of  *O. nigriventris* contigs (%) | 8789  (56.1) | 9978  (63.6) | 11420  (72.8) | 12243  (78.1) | 12819  (81.8) | 14189  (90.5) | 15107  (96.3) | 15439  (98.5) | 15585  (99.4) |
|  |  |  |  |  |  |  |  |  |  |
| **Number of reads** | **≤5000** | **≤10000** | **Total** | **Largest** |  |  |  |  |  |
| Number of *T. dichtomus*  contigs (%) | 4892  (99.9) | 4895  (99.9) | 4898  (100.0) | 45032 |  |  |  |  |  |
| Number of  *O. nigriventris* contigs (%) | 15675  (99.9) | 15679  (99.9) | 15680  (100.0) | 11416 |  |  |  |  |  |

Additional file 5. Cumulative frequencies of contig depth for *T. dichotomus* and *O. nigriventris* transcriptome assemblies.
